# Predictive and Prognostic Biomarkers in Patients With Mycosis Fungoides and Sézary Syndrome (BIO-MUSE): Protocol for a Translational Study

**DOI:** 10.2196/55723

**Published:** 2024-04-04

**Authors:** Emma Belfrage, Sara Ek, Åsa Johansson, Hanna Brauner, Andreas Sonesson, Kristina Drott

**Affiliations:** 1 Division of Dermatology and Venereology, Department of Clinical Sciences Lund University Skåne University Hospital Lund Sweden; 2 Department of Immunotechnology Faculty of Engineering Lund University Lund Sweden; 3 Clinical Genetics and Pathology, Division of Hematology and Transfusion Medicine, Department of Laboratory Medicine Skåne University Hospital Lund Sweden; 4 Division of Dermatology and Venereology, Department of Medicine and Center for Molecular Medicine Karolinska Institutet Stockholm Sweden; 5 Department of Dermatology and Venereology Karolinska University Hospital Stockholm Sweden; 6 Department of Hematology and Transfusion Medicine Skåne University Hospital Lund Sweden

**Keywords:** mycosis fungoides, Sézary syndrome, prognostic, predictive, protocol, translational study, cutaneous T-cell lymphomas (CTCL), skin microbiota, immunology, tissue microenvironment, epigenetics, quality of life, skin infection, Staphylococcus aureus, progression of disease, skin barrier, prognostic biomarkers, adult, adults, elderly, spatial, microbiological sampling, blood, study protocol

## Abstract

**Background:**

Cutaneous T-cell lymphoma (CTCL) is a rare group of lymphomas that primarily affects the skin. Mycosis fungoides (MF) is the most common form of CTCL and Sézary syndrome (SS) is more infrequent. Early stages (IA-IIA) have a favorable prognosis, while advanced stages (IIB-IVB) have a worse prognosis. Around 25% of patients with early stages of the disease will progress to advanced stages. Malignant skin-infiltrating T-cells in CTCL are accompanied by infiltrates of nonmalignant T-cells and other immune cells that produce cytokines that modulate the inflammation. Skin infection, often with *Staphylococcus aureus,* is frequent in advanced stages and can lead to sepsis and death. *S. aureus* has also been reported to contribute to the progression of the disease. Previous reports indicate a shift from Th1 to Th2 cytokine production and dysfunction of the skin barrier in CTCL. Treatment response is highly variable and often unpredictable, and there is a need for new predictive and prognostic biomarkers.

**Objective:**

This prospective translational study aims to identify prognostic biomarkers in the blood and skin of patients with MF and SS.

**Methods:**

The Predictive and Prognostic Biomarkers in Patients With MF and SS (BIO-MUSE) study aims to recruit 120 adult patients with MF or SS and a control group of 20 healthy volunteers. The treatments will be given according to clinical routine. The sampling of each patient will be performed every 3 months for 3 years. The blood samples will be analyzed for lactate dehydrogenase, immunoglobulin E, interleukins, thymus and activation-regulated chemokine, and lymphocyte subpopulations. The lymphoma microenvironment will be investigated through digital spatial profiling and single-cell RNA sequencing. Microbiological sampling and analysis of skin barrier function will be performed. The life quality parameters will be evaluated. The results will be evaluated by the stage of the disease.

**Results:**

Patient inclusion started in 2021 and is still ongoing in 2023, with 18 patients and 20 healthy controls enrolled. The publication of selected translational findings before the publication of the main results of the trial is accepted.

**Conclusions:**

This study aims to investigate blood and skin with a focus on immune cells and the microbiological environment to identify potential new prognostic biomarkers in MF and SS.

**Trial Registration:**

ClinicalTrials.gov NCT04904146; https://www.clinicaltrials.gov/study/NCT04904146

**International Registered Report Identifier (IRRID):**

DERR1-10.2196/55723

## Introduction

### Mycosis Fungoides and Sézary Syndrome

Cutaneous T-cell lymphoma (CTCL) is a rare group of lymphomas that primarily affects the skin [1-3]. The pathogenesis of CTCL is not yet fully understood [3]. The annual incidence of CTCL is 0.7 per 100,000. Mycosis fungoides (MF) is the most prevalent form and comprises 60% of CTCL, with a higher male incidence (1.6:1.0) and a peak age incidence between 50 and 74 years [4]. Since MF often has a chronic course, its prevalence is considerably higher. The skin lesions in MF can be patches, plaques, tumors, or erythroderma. Sézary syndrome (SS) is an aggressive leukemic variant of the disease and constitutes only 5% of CTCL; it is defined as a triad of erythroderma, lymphadenopathy, and the presence of neoplastic T-cells in peripheral blood [5]. The extent of the disease in the skin is measured according to the Modified Severity-Weighted Assessment Tool [6,7]. Both MF and SS are staged according to the same tumor-node-metastasis-blood (TNMB) classification, assessing the involvement of skin, peripheral lymph nodes, peripheral blood, and visceral organs [8].

### Prognosis of MF and SS

The TNMB stage at diagnosis remains the most important prognostic factor [9,10]. Many patients with early stages of MF have an indolent disease with a 5-year disease-specific survival of 89% to 98% [8,11]. However, approximately 25% of patients with early stages of disease will later progress to advanced stages [4]. Patients with advanced stages of MF have a 5-year disease-specific survival of 18% to 56% associated with treatment failure [8,10]. The 5-year disease-specific survival of SS is 36% [3,8,10].

Multivariate analyses of cohorts of patients with MF and SS have identified potential adverse prognostic factors in the early stages of disease consisting of male gender, aged >60 years, plaques, folliculotropic disease, and stage N1/NX and negative prognostic factors for advanced disease consisting of male gender, aged >60 years, stages B1/B2 and N2/N3, and visceral involvement [12,13]. The presence of plaque lesions in the early stage of the disease, large cell transformation in the skin, and elevated lactate dehydrogenase are also identified as adverse prognostic factors [11,14,15]. Still, there is an unmet clinical need to identify which patients will progress to an advanced stage of disease, and there is a great need for new reliable prognostic markers.

### Treatments for MF and SS

The therapeutic options for MF and SS range from skin-directed therapy (SDT) to systemic treatment, and the selection of appropriate treatment is primarily based on the stage of the disease. There are only a few randomized controlled trials for the treatment of MF and SS, and current recommendations for treatment are mainly based on consensus meetings and international guidelines [4,5,10]. The treatment of the early stages of MF aims at modulating the immune response in the skin through SDT, such as topical corticosteroids and UV light therapy. Local radiation therapy usually has a good effect in all stages. In early stages that are refractory to SDT or in advanced stages, systemic treatment is used, including retinoid derivatives, low-dose methotrexate, and interferon alfa. In patients with lymphoma cells expressing CD30, targeted chemotherapy with brentuximab vedotin can be used. Further, standard chemotherapy such as gemcitabine or doxorubicin can be used; however, this often results in only short remission periods. Extracorporeal photopheresis can be used in erythrodermic stages of MF and SS and mogamulizumab and alemtuzumab can be used in advanced stages of disease. Highly selected patients with advanced stages of disease can be considered for allogeneic stem cell transplantation. Histone deacetylase (HDAC) inhibitors such as vorinostat and romidepsin are approved by the US Food and Drug Administration but are not commonly used in Europe [16,17]. Total skin electron beam therapy can be used in widespread disease in the skin or before allogeneic stem cell transplantation. Treatment response is unpredictable, and there is an unmet clinical need to find reliable predictive markers.

### Immunological Changes of the Microenvironment of CTCL in Skin and Blood

Early stages of CTCL derive from mature CD4+ T-cells, or rarely CD8+ T-cells, in the skin. The restriction to the skin suggests that the affected cells are dependent on the specific cutaneous microenvironment, including cytokines and adhesion molecules. Malignant skin-infiltrating cells are accompanied by infiltrates of nonmalignant T-cells and other immune cells. The infiltrating benign immune cells produce a variety of cytokines that modulate cutaneous inflammation and are important constituents of the local environment of the tumor, fostering proliferation, survival, and migration [3]. Attempts to associate a unique cytokine profile of the disease based on skin or blood samples have indicated that a shift from Th1 to Th2 cytokine production accompanies disease progression [18,19], but the mechanisms behind this shift are not completely known [20]. In the advanced stages of the disease, a reduction of skin-infiltrating CD8+ T-cells has been observed, as well as a shift toward M2 polarized macrophages and increased frequencies of NK cells and B-cells [21,22]. Other major immunological changes also facilitate the environment of the lymphoma cells and impair the host immunological defense [22]. The hypothesis of this study is to further explore these immunological changes through analysis of lymphocyte subpopulations in blood and skin together with cytokine expression in plasma through protein array analysis. We also hypothesize that we will detect other cells, molecules, and factors in the microenvironment of the tumor that could influence the clinical course of CTCL.

### Epigenetic Modifications in CTCL

Regulation of DNA transcription is complex, and the mechanisms of regulation of DNA transcription in CTCL are partially unknown. HDACs can regulate the expression of genes and activities of transcription factors involved in malignant transformation and immune defense. HDACs act mainly through alteration of the structural components of chromatin by histone deacetylation, thus affecting the 3D conformation of DNA without changing or interrupting its sequence. Epigenetic drugs such as HDAC inhibitors have shown promising results in patients with advanced disease, emphasizing the impact of epigenetic regulators on the disease. Based on phase II data demonstrating long-term remission in 35% of heavily pretreated patients, HDAC inhibitors have been approved by the US Food and Drug Administration for second-line treatment of CTCL [16,17]. Although the dysregulated function of HDAC inhibitors may have an important role in malignant transformation and in the response to cancer treatment, the in vivo mechanisms behind the effects of HDAC inhibitors in CTCL are largely unknown. However, next-generation sequencing data have shown amino-acid or copy number alterations of 13 chromatin modifiers in 1% to 7% of CTCL cases, respectively, suggesting a mutation-driven epigenetic imbalance in CTCL [23]. Interestingly, HDAC inhibitors have been shown to reprogram the epigenome of host nonmalignant T-cells toward normal patterns, emphasizing the role of the host T-cells for therapy response [24,25].

### Microbiological Changes and Skin Barrier Function in MF and SS

Skin infections, often with *Staphylococcus aureus*, are frequently seen in advanced stages of MF and SS. In patients who are immunocompromised, skin infections can lead to sepsis and death. Some studies indicate that a shift from Th1 to Th2 cytokine production and dysfunction of the skin barrier contribute to skin infections [26,27]. The underlying basis for the microbiological changes and the skin barrier function in CTCL need to be further elucidated for their potential role in the pathogenesis and progression.

### Goals of This Study

The primary objective of this study is to identify serum-protein markers for 6-month progression-free survival (PFS) and to identify the immune cell profile in blood for 6-month PFS.

Secondary objectives are to identify the immune cell profile in the skin for 6-month PFS, analyze the lymphoma microenvironment in the skin for 6-month PFS, identify the skin barrier and skin microbiology profiles for 6-months PFS, and identify the epigenetic changes in lymphoma T-cells and host T-cells for 3 months PFS.

## Methods

### Study Design

**Figure 1:**
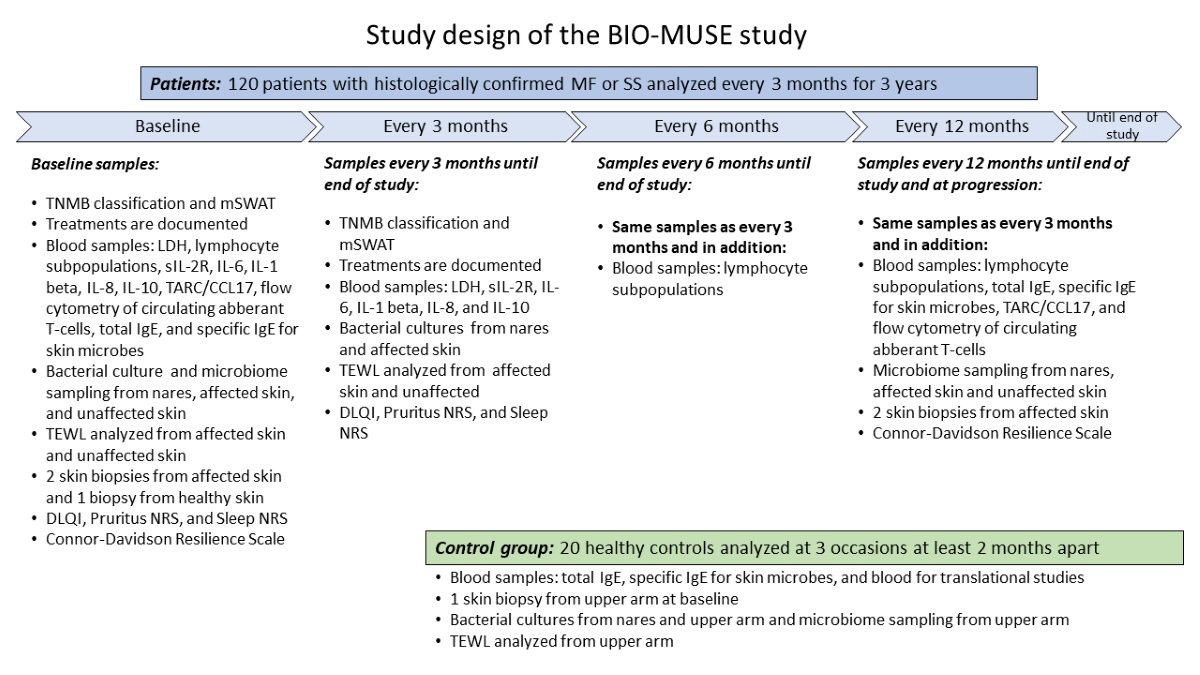
Study design of the BIO-MUSE study. BIO-MUSE: Predictive and Prognostic Biomarkers in Patients With Mycosis Fungoides and Sézary Syndrome; CT: computed tomography; DLQI: Dermatology Life Quality Index; IgE: immunoglobulin E; IL-6: interleukin-6; IL-8: interleukin-8; IL-10: interleukin-10; IL-1beta: interleukin-1beta; LDH: lactate dehydrogenase; MF: mycosis fungoides; mSWAT: Modified Severity-Weighted Assessment Tool; NRS: Numerical Rating Scale; sIL-2R: soluble interleukin-2 receptor; SS: Sézary syndrome; TARC: thymus and activation-regulated chemokine; TEWL: transepidermal water loss; TNMB: tumor-node-metastasis-blood.

The Predictive and Prognostic Biomarkers in Patients With MF and SS (BIO-MUSE) study is a translational prospective study, aiming to include 120 adult patients with MF and SS and a control group of 20 healthy volunteers (Figure 1). Sampling of each patient will be performed every 3 months for 3 years. The control group will be examined on 3 different occasions at least 2 months apart. This study is presently open at Skåne University Hospital and aims to open at Karolinska University Hospital. The multicenter study design enables other Swedish sites to be included, after further ethical approval.

### Ethical Considerations

This study’s protocol is written per the Declaration of Helsinki and the International Council for Harmonisation’s good clinical practice guidelines. This study was registered with ClinicalTrials.gov (NCT04904146) and approved by the Swedish Ethics Committee (2019-05130). Written informed consent will be obtained from participants according to the Declaration of Helsinki and International Council for Harmonisation’s good clinical practice guidelines.

### Eligibility Criteria

Inclusion criteria will be patients aged 18-100 years with histologically confirmed MF and SS (stage IA-IVB) and World Health Organization performance status 0-3. The inclusion criteria for the healthy control group will be healthy adults aged 18-100 years with World Health Organization performance status 0-2 and the absence of any malignant, autoimmune, or infectious disease. The exclusion criteria for both groups are psychiatric illnesses or conditions that could interfere with the ability to understand the requirements of this study.

### Stage of Disease and Modified Severity-Weighted Assessment Tool

Patients will be staged according to the TNMB classification [8]. The extent of disease in the skin will be measured according to the Modified Severity-Weighted Assessment Tool [6,7]. Progression or regression will be defined as a shift from one stage to another.

### Treatment of MF and SS

The treatments will be given according to clinical routine and national and international guidelines for MF and SS and participation in this study will not affect the choice of treatments. Ongoing treatments will be documented.

### Blood Samples for Soluble Interleukin-2 Receptor, Interleukins, and Thymus and Activation-Regulated Chemokine/CCL17

Blood samples in patients will be analyzed for complete blood count, liver and kidney function, lactate dehydrogenase, soluble interleukin-2 receptor, interleukin-6, interleukin-8, interleukin-10, and thymus and activation-regulated chemokine/CCL17 (Multimedia Appendix 1).

### Blood Samples for Analysis of Immunoglobulin E Against Specific Antigen

Total immunoglobulin E and specific immunoglobulin E for *S. aureus* enterotoxin A (m80), enterotoxin B (m81), toxic shock syndrome toxin-1 (Rm226), *Malassezia* (m227), and *Candida albicans* (m5) will be analyzed in patients and in the control group (Multimedia Appendices 1 and 2, respectively).

### Blood Samples for Analysis of Lymphocyte Subpopulations

Flow cytometry of peripheral blood will be performed in patients to analyze the T-cell subpopulations including naive, effector, and memory T-cells (including Th1, Th2, and Th17 cells) and activated and regulatory T-cells. Monoclonal antibodies recognizing CD4, CD8, CD197, CD45RA, CD25, CD194, CD127, CD45RO, CD183, and CD196 will be used [28]. In addition, to evaluate the presence of clonal T-cells in peripheral blood and biopsies the following panel: T-cell receptor fluorescein isothiocyanate, TRBC1 phycoerythrin, CD16 electron coupled dye, CD2 PC7, CD3 BV421, CD4PC5.5, CD7 A700, CD8 KrO, CD26 allophycocyanine, and CD45 allophycocyanine-H7 will be used [29] (Multimedia Appendices 1 and 3, respectively).

### Serum-Based Profiling of Immune-Related Soluble Proteins

Broad sets of serum-based protein profiling focused on immune-related proteins will be performed in patients. Global protein profiling will be performed using commercially available methods, allowing high-plex analyses of immune-related soluble molecules. State-of-the-art bioinformatic tools will be applied for preprocessing and analysis of data (Multimedia Appendix 1).

### Assessment of Tumor Microenvironment by Digital Spatial Profiling and Single-Cell RNA Sequencing

To allow the study of the cellular tumor immune microenvironment, 2 biopsies from affected skin and 1 biopsy from unaffected skin, at least 5 cm away from affected skin, will be taken. In the control group, 1 skin biopsy is taken from the upper arm. The skin biopsies will be formalin-fixed and paraffin-embedded. In addition, blood will be drawn from patients and healthy controls and peripheral blood mononuclear cells will be cryopreserved.

Multiplex analyses of samples taken before and after progression have the potential to contribute to novel biological knowledge as previously discussed in a recent review by the group Kalliara et al [30] and will be performed in samples collected over time. In brief, in skin biopsies, Digital Spatial Profiling will be performed to study immune infiltration in CTCL and how it evolves during the progression of the disease [31]. Single-cell RNA and T-cell receptor sequencing will be applied to sorted CD3+ cells to follow the evolution of the malignant clone over time. Moreover, additional assessments of genetic and epigenetic changes in the tumor and tumor microenvironment will be performed in skin biopsies (Multimedia Appendix 1).

### Assessment of Epigenetic Factors in Circulating Tumor Cells Compared to Normal and Host T-Cells

Cell sorting of CD3+ cells from peripheral blood in patients with blood involvement will be performed using FACSAria (BD, Bioscience). When possible, 2-3 million cells will be sorted from each patient to allow the preparation of DNA for epigenetic analysis and RNA for gene expression analysis. Analysis of histone modifications and chromatin accessibility will be performed by ChIP-seq (chromatin immunoprecipitation with sequencing) and ATAC-seq (assay of transposase-accessible chromatin with sequencing), respectively. Moreover, molecular profiling using nCounter analysis (NanoString Technologies, Inc) will be performed to profile cells through expression panels focused on immune profiling and proliferative signaling pathways (Multimedia Appendix 1).

### Microbiological Sampling

Microbiological samples will be analyzed from affected skin and from unaffected skin 5 cm away from affected skin, preferably from the upper body, and from nares in patients. In the control group, the microbiological samples will be analyzed from the upper arm and from the nares (Multimedia Appendices 1 and 2, respectively).

### Analysis of Skin Barrier Function

Skin barrier function will be measured as transepidermal water loss (TEWL) with a closed chamber TEWL meter, VapoMeter 300 (Delfin Technologies Ltd), according to guidelines [32]. TEWL will be measured on affected skin and on unaffected skin 5 cm away from affected skin, preferably from the upper body. TEWL will be measured on the upper arm in the control group (Multimedia Appendices 1 and 2).

### Hematopathology of Skin, Lymph Node, Bone Marrow, and Tumor Biopsies

Hematopathology of skin biopsy will be performed in affected skin from patients and is optional from lymph nodes, bone marrow, and tumors (Multimedia Appendices 1 and 3).

### Patient-Oriented Life Quality Measures

Patient-oriented life quality measures will be performed. The Dermatology Life Quality Index [33], Peak Pruritus Numerical Rating Scale [34], Sleep Numerical Rating Scale, and Connor-Davidson Resilience Scale [35] will be used (Multimedia Appendix 1).

### Statistical Analysis

The sample profiles from patients with an early stage of disease (stage IA-IIA) will be compared to samples from patients with an advanced stage of disease (stage IIB-IVB) using multivariate analysis with principal component analysis. Individual samples will be compared between the groups using the Mann-Whitney *U* test. Other translational samples will be presented by descriptive statistics. Progression-free and overall survival will be presented using Kaplan-Meier plots.

## Results

Patient inclusion started in 2021 and is still ongoing in December 2023, with 18 patients and 20 healthy controls enrolled. Until December 2023, a total of 2 included patients have declined further participation due to the extra visits in this study. Further, 2 patients have died from MF during this study. The publication of selected translational findings before the publication of the main results of the trial is accepted, and data analysis is in progress.

The evaluation of the stage of the disease will be observed every 3 months according to the TNMB classification, where the shift from one stage to another implies progression or treatment response. After the study, patients will be grouped as either early stage of the disease (IA-IIA) or advanced stage of the disease (IIB-IVB). The patients crossing over from the early stage of the disease to the advanced stage of the disease group will be analyzed both individually and as a group. Approximately 30% (36/120) of patients are estimated to be in the advanced stage of the disease group. The translational samples in the early stage of the disease group will be compared to samples from the advanced stage of the disease group. The treatments directed against MF are documented throughout this study. Excluded patients will be presented along with the final results. Depending on patient numbers, treatment data will be presented descriptively, or if possible, to support data on treatment predictive biomarkers.

## Discussion

### Principal Findings

This study aims to find potential predictive and prognostic biomarkers in patients with MF and SS. This study will investigate translational samples from skin and blood concerning the stage of the disease over time, which has the advantage of monitoring patients with consecutive samplings to be able to capture changes before and during a possible progression. This study will be able to compare differences in patients with early (IA-IIA) and advanced (IIB-IVB) stages of the disease. It will also be possible to analyze the data separately for patients with MF and SS, respectively. The translational samplings in this study are chosen to be able to detect changes in the immune system and in tumor cells, as well as changes in the skin barrier function and the skin microbiota. There is a clinically unmet need to find new predictive and prognostic biomarkers in MF and SS, and studies further exploring these areas are therefore a high priority. The research team, techniques, and management presented in this protocol have capacities to investigate new aspects and bring new knowledge to the field of CTCL. This study combines a multidisciplinary outpatient clinic with extensive translational research sampling, which has considerable potential to lead to true patient benefit.

### Limitations

MF and SS are rare diseases, and the inclusion of the proposed number of patients in this study protocol may take considerable time. To reach the estimated number of patients in this study, inclusion will also start at Karolinska University Hospital, and to expand this study further, cooperations with other centers is ongoing. Another possible limitation is that the control group will be followed for a shorter period compared to the patients.

### Comparison With Prior Work

The Prospective Cutaneous Lymphoma International Prognostic Index study is an ongoing international study of a confirmed early-stage MF cohort, which is being followed up to identify prognostic factors in the hope of providing better management and improving survival by identifying patients at risk of disease progression [36].

### Conclusions

The BIO-MUSE study is a prospective translational study aiming to identify new prognostic and predictive biomarkers in blood and skin in patients with MF and SS.

## Appendix

Multimedia Appendix 1 Translational samples and logistics for patients in the Predictive and Prognostic Biomarkers in Patients With Mycosis Fungoides and Sézary Syndrome (BIO-MUSE) study.

Multimedia Appendix 2 Translational samples and logistics for the healthy control group in the Predictive and Prognostic Biomarkers in Patients With Mycosis Fungoides and Sézary Syndrome (BIO-MUSE) study.

Multimedia Appendix 3 Planned assessments of hematopathology in the skin, lymph node, bone marrow, and tumor biopsies.

## Abbreviations

ATAC-seq: assay of transposase-accessible chromatin with sequencing
BIO-MUSE: Predictive and Prognostic Biomarkers in Patients With Mycosis Fungoides and Sézary Syndrome
CBC: complete blood count
ChIP-seq: chromatin immunoprecipitation with sequencing
CTCL: cutaneous T-cell lymphoma
HDAC: histone deacetylase
MF: mycosis fungoides
PFS: progression-free survival
SDT: skin-directed therapy
SS: Sézary syndrome
TEWL: transepidermal water loss
TNMB: tumor-node-metastasis-blood

## Data Availability

The data sets analyzed during this study are available from corresponding authors upon reasonable request.
