# Supplementary material for: Predictive and Prognostic Biomarkers in Patients With Mycosis Fungoides and Sézary Syndrome (BIO-MUSE): Protocol for a Translational Study
Source: JMIR Res Protoc. 2024 Apr 4;13:e55723. doi: 10.2196/55723 (PMC11027051; doi:10.2196/55723)
Supplement: Multimedia Appendix 1 [file resprot_v13i1e55723_app1.docx]

**Multimedia Appendix 1.** Translational samples and logistics for patients in the predictive and prognostic biomarkers in patients with mycosis fungoides and Sézary syndrome (BIO-MUSE) study.

|  | **Baseline** | **Every 3 months** | **Every 6 months** | **Every 12 months** | **At progression** | **Analysis performed at** |
| --- | --- | --- | --- | --- | --- | --- |
| **Blood samples** |  |  |  |  |  |  |
| CBC, lymphocytes, neutrophils | ✓ | ✓ | ✓ | ✓ | ✓ | Dept. of Clinical Chemistry; SUS/KUS |
| INR | ✓ |  |  |  |  | Dept. of Clinical Chemistry, SUS/KUS |
| Sodium, potassium, calcium, creatinine, bilirubin, AST, ALT, ALP, pancreas amylase, albumin, LDH | ✓ | ✓ | ✓ | ✓ | ✓ | Dept. of Clinical Chemistry, SUS/KUS |
| Lymphocyte subpopulations | ✓ |  | ✓ | ✓ | ✓ | Dept. of Clinical Immunology, SUS/KUS |
| sIL-2R, IL-6, IL-1 beta, IL-8, IL-10, TARC/CCL17 | ✓ | ✓ | ✓ | ✓ | ✓ | Dept. of Clinical Immunology, SUS/KUS |
| In applicable females: pregnancy test | ✓ |  |  |  |  | Dept. of Clinical Chemistry, SUS/KUS |
| Total IgE and specific IgE for skin microbes | ✓ |  |  | ✓ | ✓ | Dept. of Clinical Immunology, SUS/KUS |
| 3 vials of peripheral blood stored as whole blood, plasma, PBMCs or sorted T-cells | ✓ | ✓ | ✓ | ✓ | ✓ | Dept. of Immunotechnology, Lund University/Dept. of Medicine, KI |
| 2 ml of serum | ✓ | ✓ | ✓ | ✓ | ✓ | Dept. of Immunotechnology, Lund University/Dept. of Medicine, KI |
| **Pathological sampling** |  |  |  |  |  |  |
| 4 mm punch biopsy of affected skin (paraffin-embedded) | ✓ |  |  | ✓ | ✓ | Dept. of Pathology, SUS/KUS |
| 4 mm punch biopsy of affected skin (paraffin-embedded) | ✓ |  |  | ✓ | ✓ | Dept. of Immunotechnology, Lund University/Dept. of Medicine, KI |
| 4 mm punch biopsy of unaffected skin (paraffin-embedded) | ✓ |  |  |  |  | Dept. of Immunotechnology, Lund University/Dept. of Medicine, KI |
| Flow cytometry aberrant T-cells in blood | ✓ |  |  | ✓ | ✓ | Dept. of Pathology, SUS/KUS |
| Bone marrow biopsy (stage B1b or B2) | ✓ |  |  |  | optional | Dept. of Pathology, SUS/KUS |
| Bone marrow flow cytometry (stage B1b or B2) | ✓ |  |  |  | optional | Dept. of Pathology, SUS/KUS |
| Lymph node | optional |  |  |  | optional | Dept. of Pathology, SUS/KUS |
| Tumor or visceral biopsy | optional |  |  |  | optional | Dept. of Pathology, SUS/KUS |
| **Microbiological sampling** |  |  |  |  |  |  |
| Bacterial culture from the nares | ✓ | ✓ | ✓ | ✓ | ✓ | Clinical Microbiology, SUS/KUS |
| Bacterial culture from affected skin | ✓ | ✓ | ✓ | ✓ | ✓ | Clinical Microbiology, SUS/KUS |
| Microbiome affected skin | ✓ |  |  | ✓ | ✓ | Dept. of Clinical Sciences, Lund University |
| Microbiome unaffected skin | ✓ |  |  | ✓ | ✓ | Dept. of Clinical Sciences, Lund University |
| **Skin barrier function** |  |  |  |  |  |  |
| TEWL of unaffected skin | ✓ | ✓ | ✓ | ✓ | ✓ | Performed at the outpatient clinic |
| TEWL of affected skin | ✓ | ✓ | ✓ | ✓ | ✓ | Performed at the outpatient clinic |
| **Radiology** |  |  |  |  |  |  |
| CT scan of neck, chest, abdomen (stage IIA-IV) | ✓ |  |  |  | optional | Dept. of Radiology, SUS/KUS |
| **Clinical status** |  |  |  |  |  |  |
| WHO performance status | ✓ | ✓ | ✓ | ✓ | ✓ | Evaluated at the outpatient clinic |
| TNMB classification | ✓ | ✓ | ✓ | ✓ | ✓ | Evaluated at the outpatient clinic |
| mSWAT | ✓ | ✓ | ✓ | ✓ | ✓ | Performed at the outpatient clinic |
| Ongoing treatment of MF/SS | ✓ | ✓ | ✓ | ✓ | ✓ | Documented at outpatient clinic |
| DLQI | ✓ | ✓ | ✓ | ✓ | ✓ | Performed at the outpatient clinic |
| Pruritus NRS | ✓ | ✓ | ✓ | ✓ | ✓ | Performed at the outpatient clinic |
| Sleep NRS | ✓ | ✓ | ✓ | ✓ | ✓ | Performed at the outpatient clinic |
| Connor-Davidson Resilience scale | ✓ |  |  | ✓ | ✓ | Performed at the outpatient clinic |

BIO-MUSE: predictive and prognostic biomarkers in patients with mycosis fungoides and Sézary syndrome; SUS: Skåne University Hospital; KUS: Karolinska University Hospital; KI: Karolinska Institutet; dept: department; CMM: Center for Molecular Medicine; CBC: complete blood count; INR: international normalized ratio; AST: aspartate aminotransferase; ALT: alanine aminotransferase; ALP: alkaline phosphatase; LDH: lactate dehydrogenase; sIL-2R: soluble interleukin-2 receptor; IL-1beta: interleukin 1beta; IL-6: interleukin-6; IL-8: interleukin-8; IL-10: interleukin-10; TARC/CCL17: thymus and activation-regulated chemokine/CCL17; IgE: immunoglobulin E; PBMCs: peripheral blood mononuclear cells; TEWL: transepidermal water loss; CT: computed tomography; mSWAT: modified Severity-Weighted Assessment Tool; DLQI: Dermatology Life Quality Index, NRS: numeric rating scale.
