# Supplementary material for: Predictive and Prognostic Biomarkers in Patients With Mycosis Fungoides and Sézary Syndrome (BIO-MUSE): Protocol for a Translational Study
Source: JMIR Res Protoc. 2024 Apr 4;13:e55723. doi: 10.2196/55723 (PMC11027051; doi:10.2196/55723)
Supplement: Multimedia Appendix 2 [file resprot_v13i1e55723_app2.docx]

**Multimedia Appendix 2.** Translational samples and logistics for the healthy control group in the predictive and prognostic biomarkers in patients with mycosis fungoides and Sézary syndrome (BIO-MUSE) study.

|  | **Baseline** | **Second sampling after at least 2 months** | **Third sampling after at least 2 months** | **Analysis performed at** |
| --- | --- | --- | --- | --- |
| **Blood samples** |  |  |  |  |
| Total IgE and specific IgE for skin microbes | ✓ |  |  | Dept. of Clinical Immunology, SUS/KUS |
| 3 vials of peripheral blood stored as whole blood, plasma, PBMCs or sorted T-cells | ✓ | ✓ | ✓ | Dept. of Immunotechnology, Lund University/Dept. of Medicine, KI |
| 2 ml of serum | ✓ | ✓ | ✓ | Dept. of Immunotechnology, Lund University |
| **Pathological sampling** |  |  |  |  |
| 4 mm punch biopsy from upper arm (paraffin-embedded) | ✓ |  |  | Dept. of Immunotechnology, Lund University/Dept. of Medicine, KI |
| **Microbiological sampling** |  |  |  |  |
| Bacterial culture from the nares | ✓ | ✓ | ✓ | Clinical Microbiology, SUS/KUS |
| Microbiome from upper arm | ✓ |  |  | Dept. of Clinical Sciences Lund University |
| **Skin barrier function** |  |  |  |  |
| TEWL from upper arm | ✓ | ✓ | ✓ | Performed at the outpatient clinic |
| **Clinical status** |  |  |  |  |
| WHO performance status | ✓ | ✓ | ✓ | Evaluated at the outpatient clinic |

BIO-MUSE: predictive and prognostic biomarkers in patients with mycosis fungoides and Sézary syndrome; SUS: Skåne University Hospital; KUS: Karolinska University Hospital; KI: Karolinska Institutet; dept: department; CMM: Center for Molecular Medicine; IgE: immunoglobulin E; PBMCs: peripheral blood mononuclear cells; TEWL: transepidermal water loss.
