# Supplementary material for: Predictive and Prognostic Biomarkers in Patients With Mycosis Fungoides and Sézary Syndrome (BIO-MUSE): Protocol for a Translational Study
Source: JMIR Res Protoc. 2024 Apr 4;13:e55723. doi: 10.2196/55723 (PMC11027051; doi:10.2196/55723)
Supplement: Multimedia Appendix 3 [file resprot_v13i1e55723_app3.docx]

**Multimedia Appendix 3.** Planned assessments of hematopathology of skin, lymph node, bone marrow, and tumor biopsies.

| **Method** | **Analysis** |
| --- | --- |
| Morphology | Hematoxylin-eosin |
| Immunohistochemistry | CD20, CD3, CD30, Ki67, CD4, CD8, CD7, CD5, CD1a, CD163, CD123, PD1 and PDL1 |
| PCR analysis | TCR beta and gamma, based on the EuroClonality/BIOMED-2 Concerted Action BMH4-CT98-3936. |
| Pathological examinations | Basic pathology  Folliculotropic growth  Large cell transformation  CD30+ percentage of cells  Ki-67 percentage of cells  Presence of clonal T-cell population |
| Flow cytometry | TCR γδ, FITC, TRBC1 PE, CD16 ECD, CD2 PC7, CD3 BV421, CD4PC5.5, CD7 A700, CD8 KrO, CD26 APC, CD45 APC-H7 |

PCR: polymerase chain reaction; TCR: T cell receptor; FITC: fluorescein isothiocyanate; PE: phycoerythrin; ECD: electron coupled dye; APC: allophycocyanine.
